# A campaign of mass drug administration with artemisinin-piperaquine to antimalaria in Trobriand Islands

**DOI:** 10.1016/j.pmedr.2023.102154

**Published:** 2023-02-16

**Authors:** Guoming Li, Shaoqin Zheng, Zhenyan Zhang, Yanshan Hu, Nansong Lin, Nadia Julie, Lei Shu, Liwei Sun, Hongying Zhang, Yueming Yuan, Yuan Liang, Zhengjie Yu, Wei Xie, Ridley Mwaisiga, Jacob Morewaya, Qin Xu, Jianping Song, Changsheng Deng

**Affiliations:** a Artemisinin Research Center, Guangzhou University of Chinese Medicine, Guangzhou, Guangdong, People’s Republic of China; b Institute of Science and Technology Park, Guangzhou University of Chinese Medicine, Guangzhou, Guangdong, People’s Republic of China; c Milne Bay Provincial Health Authority, Alotau, Milne Bay Province, Papua New Guinea; d The First Affiliated Hospital of Guangzhou University of Chinese Medicine, Guangzhou, Guangdong, People’s Republic of China

**Keywords:** Grid-based management, Mass drug administration, Artemisinin-piperaquine, Islands, Mixed malaria, Coverage

## Abstract

We conducted a study on the Trobriand Islands of Papua New Guinea (PNG) in 2018 to verify the safety and efficacy of the artemisinin-piperaquine (AP) mass drug administration (MDA) campaign in regions with moderate to high mixed malaria transmission. Based on the natural topography of the Trobriand Islands, 44,855 residents from 92 villages on the islands were enrolled and divided into the main and outer islands. Three rounds of MDA were conducted using grid-based management. The primary endpoint was the coverage rate. Adverse reactions, parasitemia, and malaria morbidity were the secondary endpoints. There were 36,716 people living in 75 villages on the main island, and the MDA coverage rate was 92.58–95.68%. Furthermore, 8,139 people living in 17 villages on the outer islands had a coverage rate of 94.93–96.11%. The adverse reactions were mild in both groups, and parasitemia decreased by 87.2% after one year of surveillance. The average annual malaria morbidity has decreased by 89.3% after the program for four years. High compliance and mild adverse reactions indicated that the MDA campaign with AP was safe. The short-term effect is relatively ideal, but the evidence for long-term effect evaluation is insufficient.

## 1 Introduction

The World Health Organization (WHO) reported 241 million malaria cases and 627,000 deaths worldwide in 2020, an increase of 14 million cases and 47,000 deaths since the outbreak of the Coronavirus Disease 2019 pandemic (World Health Organization, n.d.a). The population at risk for malaria in 10 Western Pacific countries was 7.62 million, with PNG having 80% of the malaria cases, and the trend has been rising since 2015 (World Health Organization, n.d.b).

PNG has a high prevalence of falciparum and vivax malaria, with malaria-related morbidity in children occurring approximately once per child per year (Senn et al., 2013). The *P. vivax* malaria cases account for approximately 6% of all cases worldwide (World Health Organization, n.d.b). PNG is an archipelago nation, and implementation conditions in the various islands are significantly affected by natural factors. Transportation is extremely difficult, power facilities unreliable, the network is inaccessible, and health facilities are inadequate. Despite this, PNG actively sought a new malaria control and prevention strategy.

As a first-line antimalarial program, artemisinin-based combination therapy (ACT) can rapidly eliminate *Plasmodium* at the erythrocytic stage (World Health Organization, 2015, Nosten and White, 2007). In PNG, treatment with dihydroartemisinin-piperaquine (DPQ) tablets for *P. falciparum* and *P. vivax* with a 42-day follow-up period showed 100 and 92.3% efficacy, respectively (Tavul et al., 2018). AP is a fourth-generation artemisinin compound having the same effect as DPQ (Song et al., 2011). The MDA program aimed to eliminate the malarial parasite from the human body to block its transmission and quickly control malaria (Maude et al., 2014). The measure and AP had been used successfully in Cambodia (Song et al., 2010), the Union of Comoros (Deng et al., 2014, Deng et al., 2018) and the São Tomé Island(Li et al., 2021). Malaria prevention via MDA has also been recommended by WHO as one of the initiatives to accelerate disease elimination in small islands (<500,000 people) with moderate transmission zones (falciparum malaria prevalence of 10% − 15%) (World Health Organization, 2017, World Health Organization Global Malaria Programme, 2015).

However, MDA effectiveness in vivax malaria-endemic areas was ambiguous, as discussed by the WHO evidence review group (World Health Organization Global Malaria Programme, 2015). A total of eight studies of MDA were reviewed by Shah et al. (Shah et al., 2021) and found that the antimalarial drug used in MDA, co-interventions, MDA coverage, and the risk of re-introduction could not be studied due to the limited number of studies. These factors may influence the effect of MDA compared to non-MDA. And more research is needed to determine the optimal target population size, methods to improve coverage, and primaquine safety (Newby et al., 2015).

Our study assumes that the MDA campaign effectively controls and prevents moderate to high mixed malaria transmission in the islands of PNG. The archipelago's Trobriand Islands were selected to conduct the study and validate the hypotheses.

## 2 Methods

### 2.1 Study area

**Fig. 1:**
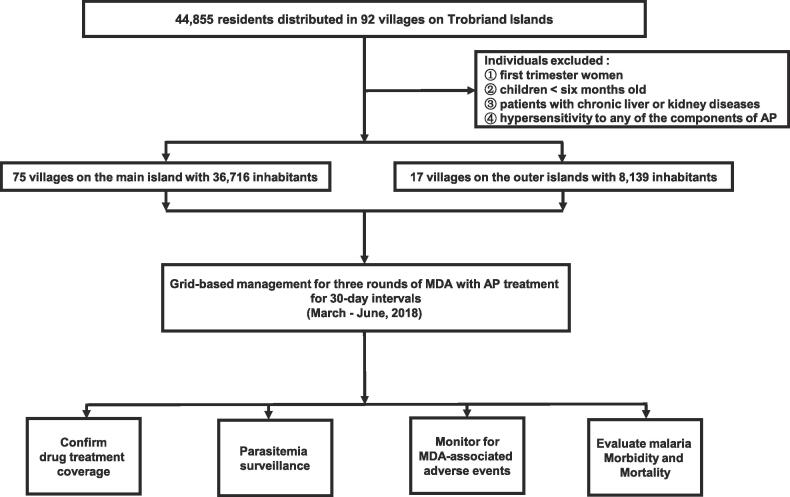
**Study profile** *AP, artemisinin-piperaquine; MDA, mass drug administration.

**Fig. 2:**
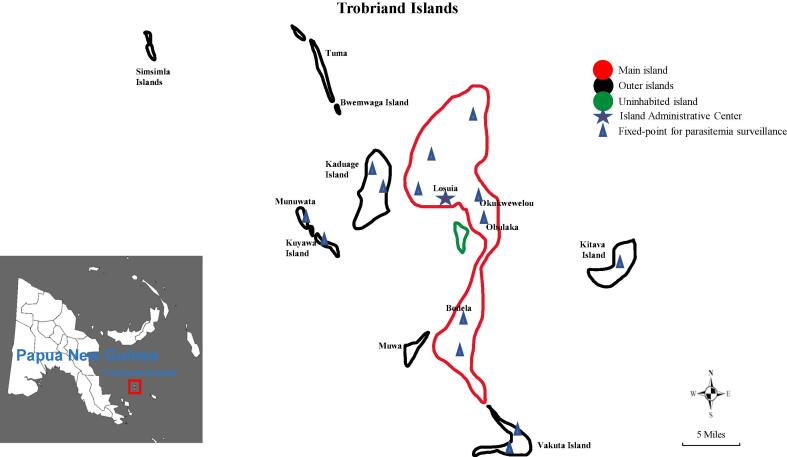
Geographic information of the Trobriand islands.

The pilot project for this study was approved by the PNG Ministry of Health (Registration Number: 20170330). Three rounds of MDA with the AP program were initiated via the grid-based management model in the Trobriand Islands from March to June 2018. The study profile is shown in Fig. 1. The Trobriand Islands were categorized into two groups in this study based on their geographical location: the main island (Kiriwina) and the outer islands (Vakuta, Kitava, Kaduaga, and Simsimla) (Fig. 2).

Trobriand Islands cover 450 km^2^ of coral atolls off the East Coast of New Guinea. Kiriwina island, located in the Trobriand Islands center, is the main island home to most of the population. This island is surrounded by the outer islands of Vakuta, Kitava, Kaduaga, and Simsimla (Wikipedia, n.d.). The rainy season in this area lasts from November to March, with the rest of the year being dry. According to a WHO report, the main malaria vectors in PNG are *Anopheles punctulatus*, *Anopheles farauti*, and *Anopheles koliensis* (World Health Organization, n.d.).

Kiriwina Rural is a long and narrow island with an area of 290.5 km^2^. According to the 2011 census, 36,721 people were living in 7,005 households distributed across 33 villages (National Statistical Office, 2011).

### 2.2 Grid-based management

This program used a grid-based management model for the division of labor and management. The management system was divided into three levels from top to bottom: experts from Guangzhou university of Chinese medicine -Milne Bay provincial health authority were allied as a central working group; health workers of Trobriand Islands for each district were constituted as a district-level working group; and grid-based managers were recruited from defined local grid residents.

Grid-based managers: two managers were recruited for each area of 200–250 people, one for implementation and the other for supervision. The specific tasks included going door-to-door in the grid-based area to 1) do community mobilization at least three times pre-MDA; 2) register the population census; 3) oral inform consent for study drugs one time pre-MDA; 4) distribute medicines and monitor the AP tablets for the entire population during MDA by observing administration and adverse reactions; 5) fill out and submit MDA data forms after each round of MDA; 6) cooperation with the team for the parasite epidemic level surveillance to conduct carrier surveys in their area. The training will include project introduction, AP treatment information, information about MDA registration forms, adverse reactions, and the corresponding paperwork completion.

The district working group is responsible for communicating information from the grid-based managers to the central working group. Each person supervises the work of 10 grid managers and has the authority to replace those who are incompetent, based on the local malaria situation as the evaluation criteria.

The central working group is mainly responsible for developing the project's antimalaria strategy and work schedule, organizing supplies and transportation, evaluating and supervising district working group personnel, as well as supervising the population carrier rate survey team and the emergency response team for adverse reactions.

### 2.3 MDA intervention

Each AP tablet had 62.5 mg artemisinin and 375 mg piperaquine and was administered as a 2-day dose regimen. Direct observation therapy (drug distribution to the individual and observed administration) was implemented for three rounds within 60 days. A full adult treatment or prophylaxis consisted of two tablets/once a day for two days in each round. The treatment or prophylaxis courses for children are given in Appendix 1.

The MDA with AP program was implemented across the entire test region. All the residents were encouraged to participate in this campaign. The exclusion criteria included age <6 months, hypersensitivity to any of the components of the drug, first-trimester women, and patients with impaired liver and renal functions.

### 2.4 Adverse reaction monitor

Previous clinical studies (Song et al., 2011, Deng et al., 2014, Deng et al., 2018) have shown that AP primarily causes headaches, dizziness, nausea, and vomiting. Participants were asked to take AP after meals. A 4-day adverse reaction monitoring was performed during each round of MDA. The residents were informed of the possible adverse reaction of AP during the door-to-door delivery by the grid managers. Residents could immediately report dizziness, nausea, vomiting, abdominal pain, diarrhea, and other symptoms to grid managers, who would record them on the adverse reaction form. Residents with mild adverse reactions were still required to complete the treatment. However, residents with severe adverse reactions would report their condition to grid managers and be allocated professional medical staff to treat it.

### 2.5 Fixed-point for parasitemia surveillance

A total of 14 villages (seven villages on the main island and seven on the outer islands) in the Trobriand Islands were selected for fixed-point monitoring of the malaria parasitemia rate based on population proportion and geographic location. Bwetalu, Kabuwaku, Kavataria, Mwatawa, Okopukopu, Okaburura, and Lalela were situated on the main island. Okayaula, Kaulak, Giva, Vakuta, Munuwata, Kuyawa, and Kasiga were situated on the outer islands. Three rounds of parasitemia monitoring were conducted in the 14 villages for one year, and samples were collected from individuals with fever, chills or any other symptoms suggestive of malaria each time.

Blood samples were collected from the patients' fingertips to prepare thick and thin blood smears. These smears were then stained with 10% Giemsa solution and examined by certified malaria microscopists. The smear was considered negative if no parasite was detected within 200 fields using an oil immersion lens (100×). About 10% of smears were randomly selected and re-examined by a second microscopist to determine the quality of the results. Any discrepancies were reviewed during the conference and resolved based on the readings of a third microscopist.

### 2.6 Statistical analyses

SPSS19.0 was used to perform statistical analyses. Qualitative indicators were described by percentage or composition ratio for descriptive statistical analyses. The mean and standard deviation describe the quantitative indicators. The Chi-square test method was used for the two groups. p < 0.05 was considered statistically significant.

## 3 Results

### 3.1 Characteristics of the study participants

Two groups implemented grid-based management within three months of MDA: 10 persons working in the central working group, 30 local health workers were trained throughout the island in a district-level working group, and 268 grid-based managers were trained to participate in this project.

A total of 44,855 residents were registered. 82% of the Trobriand Islands' population of 36,716 individuals belonging to 7,320 households were distributed in 75 villages on the main island. Furthermore, 8,139 people from 1,752 households were distributed in 17 villages on the outer islands, constituting 18% of the Trobriand Islands' population. The male-to-female ratio was 1.08:1. Moreover, the largest proportion of the population (57.74%) was adults (over 16 years of age) and amounted to 25,898 people. The proportions were similar between the two groups (p = 0.819). The smallest proportion of the population (0.87%) was infants (under six months of age), which amounted to 389 individuals. Moreover, there was no significant difference between the two groups (p = 0.938). The proportion of children (aged 6 – 24 months) on the main island was higher than on the outer islands (p = 0.001), whereas other age groups showed no significant difference (p > 0.05) (Table 1).

**Table 1:** Overall population information and MDA coverage rate for Trobriand Islands.

	Main island	Outers islands	Total	p-value
Villages-N(%)	75(81.52)	17(18.48)	92(100)	–
Households-N(%)	7320(80.69)	1752(19.31)	9072(100)	–
Male-N(%)	18988(51.72)	4270(52.46)	23258(51.85)	0.222
Age group-N(%)				
≤6 months	319(0.87)	70(0.86)	389(0.87)	0.938
6–24 months	1650(4.49)	299(3.67)	1949(4.35)	0.001
25 months-6 years	4639(12.63)	1082(13.29)	5721(12.75)	0.107
7–10 years	4000(10.89)	918(11.28)	4918(10.96)	0.315
11–15 years	4900(13.35)	1080(13.27)	5980(13.33)	0.855
≥16 years	21208(57.76)	4690(57.62)	25898(57.74)	0.819
Registered Population	36716(100)	8139(100)	44855(100)	–
Coverage rate-N(%)				
Round-1(Day 1st and 2nd)	33992(92.58)	7726(94.93)	41718(93.01)	–
Round-2(Day 31st and 32nd)	34765(94.69)	7770(95.47)	42535(94.83)	–
Round-3(Day 61st and 62nd)	35131(95.68)	7822(96.11)	42951(95.76)	–

### 3.2 MDA coverage rate

The population of the main island for the three rounds was 103,888 person-times; the population of the outer islands was 23,318 person-times, and a total of 127,206 person-times participated in MDA (Table 1). The MDA coverage rates for the three rounds were 92.58, 94.69, and 95.68% on the main island, and 94.93, 95.47, and 96.11% on the outer islands, respectively. The average coverage rate for each round was 94.5% in this program. However, 3,137, 2,320, and 1,904 people did not participate in the program in round 1, round 2, and round 3, respectively. The main reasons were travel 2,791 (2.07%), refusal 1,311 (0.97%), absence 1,072 (0.80%), serious diseases 934 (0.69%), infants (within six months), 815 (0.61%), pregnancy 345 (0.26%), and others 93 (0.07%), which accounted for about 5.5%.

### 3.3 Adverse reaction rate

A total of 2,037 (1.60%) people exhibited adverse reactions. Most adverse reactions were mild. Nausea (0.35%), vomiting (0.35%), headache (0.26%), abdominal pain (0.24%), diarrhea (0.18%), fever (0.08%), and other symptoms (0.15%) were mainly reported (Table 2), but they disappeared once the medication was discontinued.

**Table 2:** Summary of adverse events for the three-round of MDA.

Adverse Events Numbers (%*)	Main island	Outer islands	Total	p-value
Nausea	374(0.36)	72(0.31)	446(0.35)	0.538
Vomiting	387(0.37)	52(0.22)	439(0.35)	0.026
Headache	296(0.28)	40(0.17)	336(0.26)	0.064
Abdominal pain	247(0.24)	54(0.23)	301(0.24)	0.154
Diarrhea	172(0.17)	56(0.24)	228(0.18)	0.000
Fever	88(0.08)	10(0.04)	98(0.08)	0.157
Others	163(0.16)	26(0.11)	189(0.15)	0.557
Total	1727(1.66)	310(1.33)	2037(1.60)	–

*The percentage = the number of adverse events/the total population of three rounds of MDA. According to the Table 1, the population recorded for the main island for the three rounds were 33,992 + 34,765 + 35,131 = 103,888 person-times; the outer islands were 7,726 + 7,770 + 7,822 = 23,318 person-times; and the total 127,206 person-times participated in MDA.

### 3.4 Parasitemia and malaria cases surveillance

At pre-MDA, 6 months post-MDA, and 12 months post-MDA, the parasitemia rates were 14.81(36/243), 4.72(33/699), and 2.00% (14/700) on the main island and 18.40 (90/489), 2.00 (14/700), and 2.43%(17/700) on the outer islands, respectively (Table 3).

**Table 3:** Parasitemia surveillance for one year.

District N(%)	Pre-MDA						6 months post-MDA						12 months post-MDA					
	Samples	Positive					Samples	Positive					Samples	Positive				
		P.f	P.v	Mix	Pf.G	Total		P.f	P.v	Mix	Pf.G	Total		P.f	P.v	Mix	Pf.G	Total
Main island	243	31 (12.76)	3 (1.23)	2 (0.82)	6 (2.47)	36 (14.81)	699	26 （3.72)	6 （0.86)	1 (0.14)	16 (2.29)	33 (4.72)	700	12 (1.71)	2 (0.29)	0 (0)	3 (0.43)	14 (2.00)
Outer islands	489	54 (11.04)	33 (6.75)	3 (0.61)	12 (2.45)	90 (18.40)	700	11 （1.57)	3 （0.43)	0 (0)	7 (1.00)	14 (2.00)	700	14 (2.00)	2 (0.29)	1 (0.14)	10 (1.43)	17 (2.43)
Total	732	85 (11.61)	36 (4.92)	5 (0.68)	18 (2.46)	126 (17.21)	1399	37 (2.64)	9 (0.64)	1 (0.08)	23 (1.77)	47 (3.36)	1400	26 (1.86)	4 (0.29)	1 (0.07)	13 (0.93)	31 (2.21)

⁎P.f, Plasmodium falciparum; P.v, Plasmodium vivax; Mix, Plasmodium falciparum plus Plasmodium vivax; Pf.G, Plasmodium falciparum gametocyte.

**Fig. 3:**
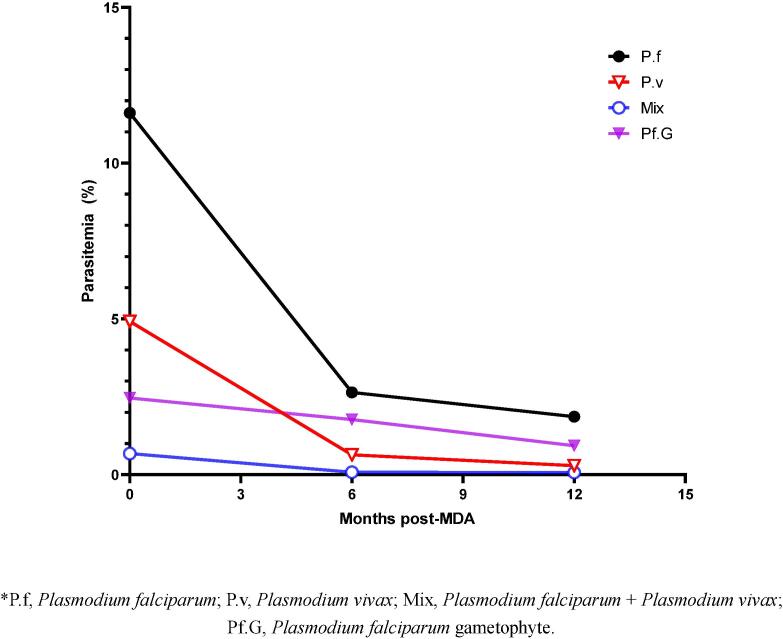
Parasitemia changed for one year.

The initial parasitemia rates for *P. falciparum*, *P. vivax*, and mixed infections were 11.61 (85/732), 4.92 (36/732) and 0.68% (5/732), respectively. These rates decreased to 2.64 (37/1399), 0.64 (9/1399) and 0.08% (1/1399) six months post-MDA, and to 1.86 (26/1400), 0.29 (4/1400), and 0.07% (1/1400) 12 months post-MDA, and the decreased rates for one year were 83.98, 94.11, and 89.71%, respectively (Table 3 and Fig. 3). The *P. falciparum* gametophytes carriage rates decreased from 2.46% pre-MDA to 1.77% after six months and 0.93% 12 months post-MDA, with a decrease of 28.05% and 62.20%, respectively (Table 3 and Fig. 3).

Malaria morbidity and mortality data of Trobriand islands for 2013 – 2021 were from the Division of Public Health for Milne Bay Provincial Health Authority, PNG. The data was derived from islandwide Medical Health Center surveillance, and did not include the fixed-point surveillance of the program. It showed that the average number of annual malaria cases from 2013 to 2017 was 1,754; however, the average number of malaria cases per year from 2019 to 2021 was 187 (an 89% decrease) after the program. The MDA campaign in 2018 was the turning point (Table 4).

**Table 4:** Malaria morbidity and mortality annual reported for Trobriand Islands (2013–2021).

Year	2013	2014	2015	2016	2017	2018	2019	2020	2021
Malaria confirmed by clinical	837	1555	1532	1113	224	127	26	35	34
Malaria confirmed by microscopy or RDT	2556	172	455	114	211	63	205	159	101
Total confirmed malaria cases	3393	1727	1987	1227	435	190	231	194	135
Malaria deaths	0	0	0	0	0	0	0	0	0

*RDT, Malaria rapid diagnostic tests.

**Data offered by local health authority, Papua New Guinea.

## 4 Discussion

We believe that MDA should be regarded in two phases: short-term and long-term effects. Regarding short-term effects, the success of antimalarial treatment depends on two theoretical factors. The first is the organizational model of MDA. How to find a better way to encourage as many people as possible to participate in MDA programs to improve the coverage of people. The second is the drug used in the campaign. The greater the drug sensitivity to the malaria parasite in the region, the more effective it is at killing the parasite in the human body.

According to the WHO recommendations, when the health system's service capacity is inadequate and is unable to supervise and treat the community epidemic promptly and effectively, MDA can be considered to reduce malaria-associated morbidity and mortality (Hetzel et al., 2018). MDA can significantly reduce malaria prevalence in malaria-endemic areas in the Solomon Islands (88.3% coverage) (Kaneko et al., 2000), Union of Comoros (85–93% coverage) (Deng et al., 2018), São Tomé Island (coverage > 98%) (Li et al., 2021), Zambia (57–70% coverage) (Fraser et al., 2022) and The Gambia (46–86% coverage) (Dabira et al., 2022). In our study, coverage in the Trobriand Islands was higher than 93% after three rounds of MDA. We summarized three possible reasons for the high coverage. Firstly, presumably grid-based management was the key to guaranteeing high coverage, demonstrated by the Union of Comoros (coverage 85–93%) (Deng et al., 2018) and São Tomé Island (coverage > 98%) (Li et al., 2021). Secondly, because of the poor living conditions on the island, the residents tend to live in a relatively concentrated area of population. Finally, the inhabitants' willingness was not to be neglected, as they have long suffered from malaria, and medicines are often unavailable to local people due to the island's natural barriers, making them value any opportunities to improve their health.

The parasitemia decreased by 87.2 % from 17.21% (pre-MDA) to 2.21% (12 months post-MDA), as inferred from the microscopic examination. The Trobriand islands were changed from moderate to high (parasite prevalence ≥ 10%) to low malaria-endemic areas (parasite prevalence < 10%). MDA could immensely reduce and maintain a low parasitemia rate and prevent the transmission of the disease during the effective monitoring period of one year, even if no proactive measures were implemented. Malaria morbidity and mortality surveillance for 2013 – 2021 revealed that, since the implementation of the project in 2018, the annual number of malaria cases on the Trobriand Islands has remained at around 200 (Table 4) in the absence of any other new measures to antimalaria even with the impact of Coronavirus Disease 2019 pandemic.

AP is a widely used in malaria treatment. Previous studies found that the detected cure rate for uncomplicated falciparum malaria was 95.1 – 100% (Trung et al., 2009, Song et al., 2011, Wang et al., 2020, Li et al., 2022), and for uncomplicated vivax malaria, it was 94.4% (Deng et al., 2008) of a 2-day dose regimen for 28- or 42-days follow-up. WHO recommends chloroquine or ACT to eliminate asexual blood parasites and 8-aminoquinoline primaquine treatment for 14 days to kill the dormant offspring and prevent a recurrence of *P. vivax* malaria (World Health Organization, 2015). However, tafenoquine and primaquine must undergo glucose-6-phosphate dehydrogenase testing before administration (Douglas et al., 2010, Sinclair et al., 2011), as they may cause hemolysis, and tafenoquine is associated with an asymptomatic decline in hemoglobin levels (Lacerda et al., 2019, Dern et al., 1955). Overall, both treatments involve certain risks. Due to the lack of medical care in remote areas and the need to reduce the population's exposure o risk, AP was used without primaquine in this study.

The standard treatment guidelines for malaria in PNG have recommended artemether-lumefantrine (AL) as the first-line treatment for *P. falciparum* malaria, AL combined with primaquine for treating *P. vivax* malaria, and DPQ as the second-line treatment since 2009 (Epidemiology and Disease Control Division Department of Health Services, 2009). In the clinical trial of AL in PNG to treat *P. vivax* and *P. falciparum* malaria in children, 12% of *P. vivax* and 3.6% of *P. falciparum* resulted in failed clinical trials within 42 days (Senn et al., 2013). The effective rate for *P. vivax* cases of DPQ in PNG with a 42-day follow-up was 92.3%, significantly higher than that of AL (Tavul et al., 2018). Furthermore, studies have reported that the use of tafenoquine and primaquine for the treatment of *P. vivax*, with a 6-month follow-up, resulted in 62.5 and 69.6% recurrence-free probability, respectively (Lacerda et al., 2019). The present study results showed that the parasitemia rate of *P. vivax* dropped from 4.92% (36/732) pre-MDA to 0.64% (9/1399) six months post-MDA and 0.29% (4/1400) 12 months post-MDA, with decline rates of 86.99 and 94.11%, respectively. These findings suggest that the external environment may improve as the risk of transmission between humans and mosquito vectors decreases after MDA, and it may contribute to controlling *P. vivax* malaria prevalence.

The prior adverse reaction rate for AP tablets in Comoros was 0.58/1,000 (Deng et al., 2018). This was the first large-scale population administration program in PNG. The total adverse reaction rate during the three rounds of MDA was 0.16/1,000 (Table 2), indicating good tolerance.

Regarding long-term effects, it is necessary to determine whether MDA can maintain a low prevalence or even eliminate malaria. The evidence for long-term effect evaluation was insufficient in this study. Five studies from Cambodia, Laos, Myanmar (2 studies), and Vietnam evaluated the immediate effect of MDA on *P. vivax*, which was not sustained over time, from RR 0.78 (4 studies) at four to six months after MDA to RR 1.12 (5 studies) at 7 – 12 months after MDA, as it was uncertain whether the decline would be sustained over the long term due to the small number of malaria cases in the research (Shah et al., 2021). Perhaps the question will remain for a long time, and it would be arbitrary to answer it now.

There were some limitations of this study. Due to a lack of infrastructure, no relevant studies on mosquito vectors or drug resistance were conducted, and the initial economic evaluation was ignored. However, the parasitemia and morbidity data from surveillance indicate a significant benefit for people living in the Trobriand Islands with limited health-care resources.

## 5 Conclusion

The MDA campaign with AP safely and effectively controlled the mixed malaria infection in the Trobriand Islands for one year, with a high compliance rate and a mild adverse reaction. The short-term effect is relatively ideal, but the evidence for long-term effect evaluation is insufficient.

## 6 Declarations

***Funding:*** This work was supported by the Natural Science Foundation of China [Grant Numbers 81873218 and 82074301], the Science and Technology Project of Guangdong Province [Grant Numbers 2021A0505030060 and 2020A0505090009], and the Chinese Medicine Cooperation Special Program of the National Administration of Traditional Chinese Medicine (Grant Number GZYYGJ2021).

***Ethical approval statement:*** This study was approved by the Department of Health of PNG (Registration Number: 20170330).

## CRediT authorship contribution statement

**Guoming Li:** Data curation, Project administration, Writing – review & editing, Writing – original draft. **Shaoqin Zheng:** Formal analysis, Supervision, Software, Visualization. **Zhenyan Zhang:** Project administration, Supervision, Investigation. **Yanshan Hu:** Investigation, Resources. **Nansong Lin:** Project administration, Resources. **Nadia Julie:** Project administration, Resources. **Lei Shu:** Project administration, Resources. **Liwei Sun:** Formal analysis. **Hongying Zhang:** Resources. **Yueming Yuan:** Resources. **Yuan Liang:** Resources. **Zhengjie Yu:** Investigation. **Wei Xie:** Investigation. **Ridley Mwaisiga:** Project administration, Resources. **Jacob Morewaya:** Project administration, Resources. **Qin Xu:**Writing – review & editing. **Jianping Song:** Conceptualization, Funding acquisition, Writing – review & editing. **Changsheng Deng:** Methodology, Funding acquisition, Supervision, Validation, Writing – review & editing.

## Declaration of Competing Interest

The authors declare that they have no known competing financial interests or personal relationships that could have appeared to influence the work reported in this paper.

## Data Availability

Data will be made available on request.
