# Supplementary material for: A campaign of mass drug administration with artemisinin-piperaquine to antimalaria in Trobriand Islands
Source: Prev Med Rep. 2023 Feb 16;32:102154. doi: 10.1016/j.pmedr.2023.102154 (PMC9958052; doi:10.1016/j.pmedr.2023.102154)
Supplement: Supplementary data 1 [file mmc1.docx]

| **Table A1. Treatment course with AP** | |
| --- | --- |
| **Age (years)** | **Dosage for per round of MDA** |
| ≥16 | 2 tablets per day for 2 days |
| 11 – 15 | 1½ tablets per day for 2 days |
| 7 – 10 | 1 tablet per day for 2 days |
| 3 – 6 | ¾ tablet per day for 2 days |
| 1/2–2 | ½ tablet per day for 2 days |

*AP, artemisinin-piperaquine; MDA, mass drug administration.
